# Comparison of clinical efficacy of unilateral biportal endoscopic lumbar interbody fusion and osterior lumbar interbody fusion in the treatment of L4/5 lumbar disc herniation

**DOI:** 10.3389/fsurg.2025.1719911

**Published:** 2025-12-18

**Authors:** Kuoang Deng, Min Yang, Yongtao Xu

**Affiliations:** The Third Orthopedic Ward, Jingzhou Hospital Affiliated to Yangtze University, Jingzhou, Hubei, China

**Keywords:** unilateral biportal endoscopic technique, lumbar disc herniation, ULIF, PLIF, L4/5

## Abstract

**Objective:**

To compare the clinical efficacy between unilateral biportal endoscopic lumbar interbody fusion (ULIF) and traditional posterior lumbar interbody fusion (PLIF) in the treatment of L4/5 lumbar disc herniation (LDH).

**Methods:**

A total of eighty-five patients with L4/5 LDH were enrolled and assigned into two groups: the ULIF group (*n* = 37) and the PLIF group (*n* = 48). Data regarding surgical conditions, hospital stay, perioperative Visual Analogue Scale (VAS) scores, Japanese Orthopaedic Association (JOA) scores, and serum inflammatory factor levels were recorded.

**Results:**

No statistically significant differences were observed in preoperative clinical characteristics (including age, disease duration, BMI, gender distribution, preoperative VAS scores, JOA scores, and serum inflammatory factor levels) between the two groups (all *P* > 0.05), indicating good comparability. Compared with the PLIF group, the ULIF group was associated with significantly less intraoperative blood loss, reduced postoperative drainage volume, and a shorter postoperative hospital stay (all *P* < 0.05). Additionally, the ULIF group exhibited lower serum levels of interleukin-6 (IL-6) and C-reactive protein (CRP) at 24 h postoperatively, as well as significantly lower VAS scores and higher JOA scores at 7 and 30 days postoperatively (all *P* < 0.05). However, the ULIF group was associated with a longer operative time and a higher number of intraoperative C-arm fluoroscopies compared with the PLIF group (both *P* < 0.05).

**Conclusion:**

ULIF exhibits significant advantages in minimally invasive. Although it requires longer operative time and more intraoperative fluoroscopies, it is associated with reduced blood loss, attenuated inflammatory responses, shorter hospital stay, and superior early postoperative pain and functional recovery, facilitating patient rehabilitation.

## 1 Introduction

Simple lumbar discectomy offers a viable treatment option for pure lumbar disc herniation (1). Although it can relieve nerve root compression with minimal trauma, it is associated with the risk of postoperative recurrence and is not suitable for patients with intervertebral space stenosis or lumbar instability caused by massive disc herniation (2). PLIF can effectively address the issues of postoperative recurrence of lumbar disc herniation, intervertebral space collapse caused by massive lumbar disc herniation, and lumbar instability by thoroughly removing the herniated disc at the affected segment and promoting intervertebral fusion (3). Therefore, PLIF is an important surgical method for treating lumbar diseases, but the traditional posterior approach is associated with significant surgical trauma and delayed postopertaive recovery. In recent years, the application of unilateral biportal endoscopic technology in spinal surgery has gradually increased, and its minimally invasive characteristics have provided a new option for lumbar fusion (4). From July 2021 to March 2025, our department treated 85 patients with L4/5 lumbar disc herniation using ULIF and PLIF. This study compared the clinical efficacy of the two surgical approaches.

## 2 Materials and methods

### 2.1 General information

A retrospective analysis was conducted on 85 patients diagnosed with L4/5 LDH who underwent lumbar fusion surgery in Jingzhou Hospital Affiliated at Yangtze University from July 2021 to March 2025. Among them, 37 patients were treated with ULIF and 48 patients were treated with PLIF. The patients were assigned to the ULIF group (*n* = 37) and the PLIF group (*n* = 48) according to the surgical methods.

### 2.2 Inclusion and exclusion criteria

#### 2.2.1 Inclusion criteria

(1) Patients diagnosed with L4/5 LDH via preoperative imaging examination; (2) Patients with persistent low back and leg pain or neurological dysfunction who failed to respond to conservative treatment for more than 3 months; (3) Patients with massive lumbar disc herniation or those who had indications for lumbar fusion surgery due to other reasons; (4) During the retrospective analysis, the patients were contacted by phone, and provided informed consent for the use of their clinical data in this study. Cases that met all the above conditions could be included in this study.

#### 2.2.2 Exclusion criteria

(1) Patients diagnosed with L4/5 LDH who only underwent simple discectomy without L4/5 fusion surgery; (2) Patients who underwent L4/5 vertebral fusion surgery along with fusion of other segments, such as L3/4 or L5/S1; (3) Patients with a history of simple L4/5 discectomy, i.e., L4/5 fusion surgery was a secondary surgery; (4) Cases with special conditions that affected intraoperative blood loss or hospital stay, such as delayed surgery due to preoperative anticoagulant withdrawal, hypertension, etc.; (5) During the retrospective analysis, the patients or their family members disagreed to use the patients’ clinical data for the data analysis of this study when contacted by phone. Cases that met any of the above conditions were excluded.

### 2.3 Surgical methods

#### 2.3.1 Surgical method for the ULIF group

Following combined intravenous-inhalation anesthesia, the patient was positioned prone with protective eye patches applied. C-arm fluoroscopy was used to locate the target segment and mark it on the body surface. Routine disinfection and sterile draping were performed. Two 10 mm incisions were made 15 mm above and below the intersection of the horizontal line of the target intervertebral space and the inner edge of the pedicle. Following soft tissue dilation, the endoscopic channel and working channel were inserted, and continuous normal saline irrigation was used to maintain a clear surgical field. Subsequent steps included removal of residual soft tissues, achievement of hemostasis, and exposure of the lamina and inferior articular process. Partial lamina was then resected to expose the ligamentum flavum, followed by dissection of superior articular process to relieve nerve root compression. After retracting the nerve root, the herniated nucleus pulposus was removed. The cartilaginous endplate was curetted until cancellous bone was exposed, and autologous bone and an intervertebral fusion cage were implanted. After confirming the correct position via C-arm fluoroscopy, the endoscope was withdrawn. On the same side, pedicle screws were inserted percutaneously through the original working channel incision. When the hard guide pin penetrated the cortex to the posterior edge of the pedicle, fluoroscopy was performed to confirm that the needle tip was located at the outer edge of the pedicle. When the guide pin was advanced to the posterior edge of the vertebral body, fluoroscopy was performed again to confirm it was at the inner edge of the pedicle. After tapping, screws of appropriate length were inserted. Postoperative management included: achieving adequate hemostasis, placing bilateral drainage tubes, suturing the incision, and applying sterile dressings.

#### 2.3.2 Surgical method for the PLIF group

After general anesthesia, the patient was positioned prone with protective eye patches applied. C-arm fluoroscopy was used to locate the target segment. Routine disinfection, draping, and coverage with a sterile transparent film were performed. A 10 cm midline incision was made in the lumbosacral region, centered on the target intervertebral space. The skin and subcutaneous tissue were incised layer by layer, the lumbodorsal muscle fascia was dissected, and the bilateral laminae and articular processes were exposed. Fluoroscopy was performed again to confirm the segment. Locating pins were inserted at the pedicle entry points. Part of the laminae, articular processes, and hyperplastic tissues were removed to fully decompress the spinal canal, protect the dura mater and nerve roots, and the annulus fibrosus was incised to remove the intervertebral disc tissue. The endplate cartilage was curetted, autologous bone and an intervertebral fusion cage were implanted, and pedicle screws were inserted along the locating pins. After confirming the position via fluoroscopy, connecting rods were placeed for fixation. The wound was irrigated with normal saline, adequate hemostasis was achieved, two negative-pressure drainage tubes were placed, instruments were counted to ensure none were missing, and the incision was sutured layer by layer, followed by application of sterile dressings.

### 2.4 Evaluation indicators

#### 2.4.1 Demographics

The baseline characteristics of patients in the ULIF group and the PLIF group, such as age, gender, and disease duration, were compared.

#### 2.4.2 Surgery-related indicators

The operative time, intraoperative blood loss, postoperative drainage volume, postoperative hospital stay, and number of intraoperative C-arm fluoroscopies were compared between the ULIF group and the PLIF group.

The anesthesia records of included patients were reviewed to extract the operative time and intraoperative blood loss for the ULIF and PLIF groups; the surgical records were reviewed to record the number of intraoperative fluoroscopies per patient; the medical records and nursing records were reviewed to record the hospital stay and postoperative drainage volume of each patient.

#### 2.4.3 Biochemical indicators

The laboratory test result system was reviewed to extract the preoperative and postoperative C-reactive protein (CRP), procalcitonin (PCT), interleukin-6 (IL-6), and serum amyloid A (SAA) levels of patients in the two groups to evaluate the postoperative inflammatory status.

#### 2.4.4 Clinical efficacy indicators

In this study, the Japanese Orthopaedic Association (JOA) lumbar function score was used to assess the preoperative and postoperative lumbar function of the participants. This scale has a total score of 29 points with scores positively correlated with functional level. Scores of 0–9 points indicate severe functional limitation, 10–15 points indicate moderate limitation, 16–24 points indicate good function, and 25–29 points indicate complete functional recovery. Lower scores indicate a higher degree of lumbar function limitation. The Visual Analog Scale (VAS) was used to measure the pain intensity in patients, with scores ranging from 0 to 10 points. A score of 0 points indicates no pain, and 10 points indicates severe, unbearable pain. The JOA scores and VAS scores of each patient were extracted preoperatively, and at 1 day, 7 days, and 30 dayspostoperatively by reviewing the nursing records and conducting telephone follow-ups.

### 2.5 Statistical methods

SPSS 22.0 was used for analyses. Continuous data are mean ± standard deviation (x¯ ± s). Baseline comparisons: independent samples t-test (continuous variables), chi-square test (gender); Cohen's d for non-significant differences. Key outcomes: ANCOVA (BMI-adjusted), mixed-effects models (repeated VAS/JOA), multivariate linear regression (age/BMI-adjusted), and independent samples *t*-test were applied. *P* < 0.05 was significant; Cohen's d was reported.

## 3 Results

### 3.1 Baseline characteristics

Analysis of the baseline characteristics of the two groups revealed that no statistically significant difference in age and disease duration between the two groups (*P* > 0.05), indicating that the two groups of patients were comparable. Detailed data are shown in Table 1.

**Table 1:** Patient demographics.

Indicator		PLIF	ULIF	T/*χ*^2^	*P*
Age (years)		68.44 ± 4.7756	66.35 ± 5.4888	1.334	0.1892
Course of disease (years)		4.28 ± 1.6618	5 ± 1.9748	1.298	0.2013
BMI		24.136 ± 2.8528	23.035 ± 3.1283	1.205	0.2350
Gender	Male	28	19	0.413	0.520
	Female	20	18		

### 3.2 Surgery-Related indicators

Analysis of Covariance (ANCOVA) with BMI as a covariate was used to compare surgery-related indicators between the two groups, and results are summarized in Table 2. The ULIF group had significantly longer operative time [135.65 ± 11.73 vs. 92.16 ± 10.26 min; adjusted mean difference (AMD) = 43.49 min, 95% CI: 39.57–47.41, F = 168.06, *P* < 0.001] and more intraoperative C-arm fluoroscopies (14.24 ± 1.445 vs. 3.20 ± 0.40 times; AMD = 11.04, 95% CI: 10.62–11.46, F = 1275.10, *P* < 0.001), with extremely large effect sizes (Cohen's d = 3.72, 10.98). In contrast, the ULIF group showed substantial advantages: significantly less intraoperative blood loss (93.30 ± 9.397 vs. 180.16 ± 17.267 mL; AMD = −86.86, 95% CI: −91.54 to −82.18, F = 389.85, *P* < 0.001), lower postoperative drainage volume (75.25 ± 7.661 vs. 112.08 ± 12.109 mL; AMD = −36.83, 95% CI: −40.31 to −33.35, F = 133.82, *P* < 0.001), and shorter postoperative hospital stay (10.20 ± 1.122 vs. 12.72 ± 1.15 days; AMD = −2.52, 95% CI: −2.87 to −2.17, F = 52.10, *P* < 0.001), all with extremely large effect sizes (Cohen's d = 5.81, 3.54, 2.21).

**Table 2:** Surgery-Related information.

Indicator	PLIF group (Mean ± SD)	ULIF group (Mean ± SD)	Adjusted mean difference (ULIF—PLIF)	F value	*P* value	Effect size (Cohen's d)
Operative time (min)	92.16 ± 10.26	135.65 ± 11.73	43.49	168.06	<0.001	3.72 large effect
Intraoperative blood loss (ml)	180.16 ± 17.267	93.30 ± 9.397	−86.86	389.85	<0.001	5.81 large effect
Postoperative drainage volume (ml)	112.08 ± 12.109	75.25 ± 7.661	−36.83	133.82	<0.001	3.54 large effect
Postoperative hospital stay (days)	12.72 ± 1.15	10.20 ± 1.122	−2.52	52.10	<0.001	2.21 large effect
Number of intraoperative C-arm fluoroscopies	3.20 ± 0.40	14.24 ± 1.445	11.04	1,275.10	<0.001	10.98 large effect

### 3.3 Inflammation-Related indicators

No significant statistical differences were oberserved in preoperative 24 h procalcitonin (PCT), postoperative 24 h PCT, preoperative 24 h C-reactive protein (CRP), and preoperative 24 h interleukin-6 (IL-6) between the two groups (*P* > 0.05). The postoperative 24 h CRP, IL-6, and serum amyloid A (SAA) in the ULIF group were lower than those in the PLIF group, with statistically significant differences (*P* < 0.05). Detailed data are shown in Table 3.

**Table 3:** Inflammation-related indicators.

Indicator	PLIF	ULIF	t	*P*
Preoperative 24 h C-reactive protein (CRP)	4.116 ± 0.7336	4.27 ± 1.046	2.370	0.5742
Postoperative 24 h C-reactive protein (CRP)	29.024 ± 2.967	21.03 ± 2.993	8.746	<0.01
Preoperative 24 h procalcitonin (PCT, ng/mL)	0.104 ± 0.662	0.135 ± 0.096	1.247	0.2190
Postoperative 24 h procalcitonin (PCT, ng/mL)	0.72 ± 0.736	0.975 ± 0.432	1.341	0.1870
Preoperative 24 h serum amyloid A (SAA, mg/L)	3.112 ± 0.584	2.87 ± 0.711	1.226	0.2270
Postoperative 24 h serum amyloid A (SAA, mg/L)	61.924 ± 6.758	48.76 ± 5.745	6.778	<0.01
Preoperative 24 h interleukin-6 (IL-6, pg/mL)	5.38 ± 0.709	5.205 ± 0.603	0.859	0.3951
Postoperative 24 h interleukin-6 (IL-6, pg/mL)	39.964 ± 4.411	31.175 ± 4.962	6.140	<0.01

### 3.4 Clinical efficacy-related indicators

To account for potential confounding by baseline BMI, Analysis of Covariance (ANCOVA) was used to compare Visual Analogue Scale (VAS, pain intensity) and Japanese Orthopaedic Association (JOA, lumbar function) scores between the two groups across all time points, with BMI as a covariate (Tables 4, 5). Preoperatively and at 1 day postoperatively, VAS scores were comparable between groups (PLIF: 7.16 ± 0.731/1.64 ± 0.656; ULIF: 7.30 ± 0.954/1.65 ± 0.726). Adjusted mean differences (AMDs) were 0.14 (95% CI: −0.32 to 0.60, F = 0.30, *P* = 0.589) and 0.01 (95% CI: −0.28 to 0.30, F = 0.002, *P* = 0.963), with small/trivial effect sizes (Cohen's d = 0.16/0.02). At 7 and 30 days postoperatively, the ULIF group had significantly lower VAS scores (2.25 ± 0.698/1.15 ± 0.792 vs. PLIF: 3.12 ± 1.032/1.72 ± 0.601), with AMDs of −0.87 (95% CI: −1.42 to −0.32, F = 10.44, *P* = 0.002, d = 0.98) and −0.57 (95% CI: −1.01 to −0.13, F = 7.20, *P* = 0.009, d = 0.79).

**Table 4:** VAS scores.

TimePoint	PLIF group (mean ± SD)	ULIF group (mean ± SD)	Adjustedmean difference (ULIF-PLIF)	F value	*P* value	Effect size (Cohen'sd)
1 day preoperative	7.16 ± 0.731	7.30 ± 0.954	0.14	0.30	0.589	0.16 small effect
1 day postoperatively	1.64 ± 0.656	1.65 ± 0.726	0.01	0.002	0.963	0.02 small effect
7 days postoperatively	3.12 ± 1.032	2.25 ± 0.698	−0.87	10.44	0.002	0.98 medium effect
30 days postoperatively	1.72 ± 0.601	1.15 ± 0.792	−0.57	7.20	0.009	0.79 medium effect

**Table 5:** JOA scores.

Time point	PLIF group (mean ± SD)	ULIF group (mean ± SD)	Adjustedmean difference (ULIF-PLIF)	F value	*P* value	Effect size (Cohen'sd)
1 day preoperative	7.84 ± 0.731	8.15 ± 0.762	0.31	1.92	0.173	0.41 small effect
7 days postoperatively	12.44 ± 1.061	14.10 ± 1.044	1.66	26.35	<0.001	1.58 large effect
30 days postoperatively	18.04 ± 0.916	18.70 ± 0.954	0.66	5.31	0.026	0.70 medium effect

Preoperatively, JOA scores were similar (PLIF: 7.84 ± 0.731; ULIF: 8.15 ± 0.762; AMD = 0.31, 95% CI: −0.12 to 0.74, F = 1.92, *P* = 0.173, d = 0.41). Postoperatively, the ULIF group had significantly higher JOA scores at 7 days (14.10 ± 1.044 vs. 12.44 ± 1.061; AMD = 1.66, 95% CI: 1.12–2.20, F = 26.35, *P* < 0.001, d = 1.58) and 30 days (18.70 ± 0.954 vs. 18.04 ± 0.916; AMD = 0.66, 95% CI: 0.09–1.23, F = 5.31, *P* = 0.026, d = 0.70).

**Figure 1:**
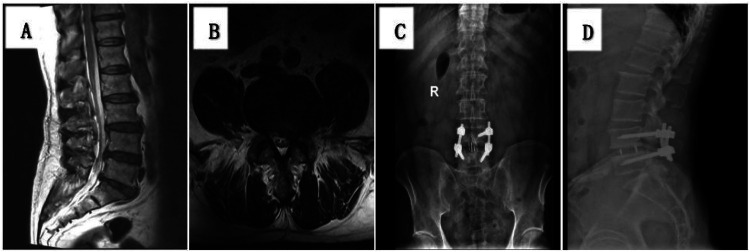
**(A,B)** preoperative MRI showing L4/5 lumbar disc herniation; **(C,D)** lumbar anteroposterior and lateral reexamination 3 days after surgery showing good position of internal fixation.

Typical case of PLIF: A 64-year-old male patient with left lower extremity radicular pain for 1 year. Lumbar MRI confirmed a diagnosis of L4/5 LDH. The patient underwent PLIF surgery, and the left lower extremity radicular pain was significantly relieved after surgery (Figure 1).

**Figure 2:**
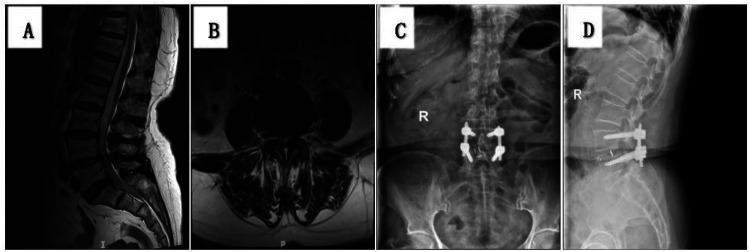
**(A,B)** preoperative MRI showing L4/5 lumbar disc herniation; **(C,D)** lumbar anteroposterior and lateral reexamination 3 days after surgery showing good position of internal fixation.

Typical case of ULIF: A 58-year-old male patient with left lower extremity radicular pain for 3 months. Lumbar MRI confirmed a diagnosis of L4/5 LDH. The patient underwent ULIF surgery (Figure 2), and the surgical outcome was good.

## 4 Discussion

### 4.1 Treatment of LDH

The treatment methods for lumbar disc herniation (LDH) are divided into conservative treatment and surgical treatment. Conservative treatment includes medication [such as non-steroidal anti-inflammatory drugs (NSAIDs) and neurotrophic drugs], physical therapy (traction, physical therapy, core muscle exercise), nerve block (epidural injection), and lifestyle adjustments (posture improvement, weight control) (5, 6). Surgical treatment is mainly indicated for patients who fail three months of conservative treatment or have neurological damage, including minimally invasive surgery (such as spinal endoscopic surgery) and open surgery (such as simple discectomy, intervertebral fusion, laminectomy decompression, or artificial disc replacement). The selection of treatment should be individualized according to the patient's condition and needs (7).

### 4.2 Characteristics of traditional PLIF surgery

Posterior lumbar decompression, bone graft fusion, and internal fixation is a surgical intervention for severe lumbar diseases. By implanting bone grafts and internal fixation devices through a posterior incision, bony fusion between vertebral bodies was achieveed, thereby resolving intractable pain and functional disorders caused by lumbar instability, nerve compression, or structural deformities (8, 9). Its core values include: (1) Reconstructing spinal stability: by fusing lumbar segments with abnormal movement, it terminates mechanical imbalance caused by intervertebral disc degeneration, spondylolisthesis, or trauma (10); (2) Nerve decompression: directly removing pathological tissues that compress the spinal cord or nerve roots, such as herniated nucleus pulposus and hyperplastic osteophytes, to relieve radicular pain in the lower limbs and sensory-motor abnormalities; (3) Deformity correction: adjusting the vertebral sequence with internal fixation devices to restore the physiological curvature and force line of the lumbar spine. However, this surgery exhibits significant limitations: (1) Adjacent segments are prone to accelerated degeneration due to stress concentration, and bone fusion failure may lead to pseudoarthrosis; (2) it is associated with intraoperative risks such as nerve injury, cerebrospinal fluid leakage, and incision infection, and permanently reduced lumbar range of motion postoperatively; (3) It exerts no beneficial effect on chronic nerve injury or non-structural pain (such as muscle strain), necessitating strict screening of surgical indications (11).

### 4.3 Core characteristics of the ULIF approach

As an innovative method in the field of spinal endoscopy that combines the advantages of open surgery and endoscopic technology (12), the derived ULIF approach exhibits significant characteristics in clinical application: (1) ULIF demonstrates prominent minimally invasive advantages. It not only does involves significantly less intraoperative blood loss and postoperative drainage than the traditional PLIF approach, but also results in lower VAS scores and higher JOA scores at 7 and 30 days postoperatively, with better short-term recovery effects. This is attributed to the superior preservation of the normal spanal anatomical structure with minimal surgical trauma; (2) The postoperative inflammation-related biochemical indicators of ULIF are significantly lower than those of PLIF. The continuous normal saline irrigation iutilized in the operation maintains a clear surgical field, enabling precise identification and hemostasis under endoscopy, and avoiding injury to surrounding tissues from electrocoagulation; (3) It integrates the efficacy of open surgery with the minimally invasive characteristics of endoscopy, providing a more favorable option for the treatment of lumbar diseases. However, ULIF also has obvious shortcomings: (1) Its total operative time is significantly longer than that of PLIF, and the operational efficiency needs further optimization; (2) The learning cost of this approach is relatively high. Beginners need to overcome the difficulty of precise tissue identification under endoscopy, resulting in a long learning curve; (3) In the initial stage, inadequate proficiency may lead to deviations in tissue identification, consequently increasing the risk of postoperative complications. (4) Osseous impingement, a common pathological feature in lumbar degenerative diseases, remains a major challenge for minimally invasive spinal procedures due to the restricted surgical access.

### 4.4 Mechanism of reduced inflammatory response in the ULIF group

This study demonstrated that patients in the ULIF group exhibited significantly lower postoperative levels of core inflammatory factors (IL-6 and CRP) compared with those in the PLIF group, which can be primarily attributed to two technical advantages inherent to the ULIF procedure.

Mechanistically, continuous normal saline irrigation during ULIF maintains the local tissue temperature at approximately 37℃, which mitigates electrocautery-induced thermal injury and subsequent tissue necrosis—a key trigger for inflammatory factor release (13). This hypothermic protective effect further inhibits the activation of the TLR4/NF-κB signaling pathway, thereby suppressing the transcription and secretion of pro-inflammatory cytokines. Consistent with prior clinical evidence, continuous irrigation has been shown to directly dilute local inflammatory exudates and reduce postoperative inflammatory responses (14).

Additionally, ULIF adopts a minimally invasive intermuscular approach via two 5 mm incisions, which maximizes the preservation of paravertebral muscle integrity. In contrast, PLIF requires extensive muscle dissection and prolonged retraction, leading to significant soft tissue injury (15). The reduced surgical trauma associated with ULIF not only alleviates surgical stress but also minimizes postoperative hematoma formation, thereby avoiding secondary inflammation induced by endogenous pyrogens released during phagocytosis of necrotic tissue. Meta-analyses have confirmed that minimally invasive spinal surgery, compared with open fusion techniques such as PLIF, consistently results in milder inflammatory responses and shorter hospital stays (16).

These findings advance our understanding of the anti-inflammatory mechanisms underlying minimally invasive spinal surgery (17), highlighting that ULIF achieves reduced postoperative inflammation through the dual effects of hypothermic irrigation and soft tissue preservation. This provides a theoretical basis for the clinical application of ULIF in improving postoperative recovery (18, 19). Acknowledging the limitations of this study—such as the lack of molecular-level detection of inflammatory pathways—future research should investigate key signaling molecules (e.g., NF-κB, p38 MAPK) to further validate and elaborate on this mechanism.

### 4.5 Research conclusions

Based on the above results, this study concludes that: In the surgical treatment of L4/5 lumbar disc herniation, compared with the traditional PLIF surgery, ULIF surgery offers significant advantages over traditional PLIF, including reducing surgical trauma, alleviating systemic inflammatory responses, and shortening the hospital stay. Although ULIF surgery requires a longer operative time and a higher number of intraoperative fluoroscopies, it offers greater advantages in terms of early postoperative recovery rate. Based on the conclusions of this study, future research and optimization directions of ULIF surgery should focus on further reducing the operative time and the number of fluoroscopies, with the goal of achieving more extensive and comprehensive application in the treatment of lumbar degenerative diseases.

This study still has limitations. Firstly, Only patients with lesions at the L4/5 segment were included, because the difficulty of percutaneous screw placement at the S1 segment is significantly higher than that at the L4 and L5 segments. Secondly, the sample size is relatively small, and more large-sample and multi-center studies are needed to verify the clinical efficacy of these two surgical methods.

## Data Availability

The original contributions presented in the study are included in the article/Supplementary Material, further inquiries can be directed to the corresponding authors.
